# The novel histone deacetylase inhibitor, N-hydroxy-7-(2-naphthylthio) hepatonomide, exhibits potent antitumor activity due to cytochrome-*c*-release-mediated apoptosis in renal cell carcinoma cells

**DOI:** 10.1186/s12885-014-1003-1

**Published:** 2015-01-23

**Authors:** Ki Cheong Park, Jun Hyeok Heo, Jeong Yong Jeon, Hye Ji Choi, A Ra Jo, Seung Won Kim, Ho Jeong Kwon, Sung Joon Hong, Kyung Seok Han

**Affiliations:** Department of Urology and Urological Science Institute, Yonsei University College of Medicine, 50-1, Yonsei-ro, Seodaemun-gu, Seoul, 120-752 Korea; Department of Surgery, Yonsei University College of Medicine, Seoul, Korea; Brain Korea 21 PLUS Project for Medical Science, Yonsei University College of Medicine, Seoul, Korea; Severance Biomedical Science Institute, Yonsei University College of Medicine, Seoul, Korea; Department of Nuclear Medicine, Yonsei University College of Medicine, Seoul, Korea; Department of Biotechnology, Translational Research Center for Protein Function Control, College of Life Science & Biotechnology, Yonsei University, Seoul, Korea

**Keywords:** HNHA, Histone deacetylase (HDAC) inhibitor, Renal cell carcinoma (RCC), Cytochrome c, Apoptosis

## Abstract

**Background:**

Epigenetic modifications play a critical role in the regulation of all DNA-based processes, such as transcription, repair, and replication. Inappropriate histone modifications can result in dysregulation of cell growth, leading to neoplastic transformation and cell death. Renal tumors have been shown to have a higher global methylation percentage and reduced histone acetylation. Preclinical models have revealed that histone gene modifiers and epigenetic alterations play important roles in renal cell carcinoma (RCC) tumorigenesis. Recently, a novel HDAC inhibitor, N-hydroxy-7-(2-naphthylthio) heptanomide (HNHA), has been introduced as an example of a new class of anti-cancer agents. The anti-cancer activity of HNHA and the underlying mechanisms of action remain to be clarified.

**Methods:**

The MTS assay using a panel of RCC cells was used to evaluate the anti-proliferative effects of HNHA. The established HDAC inhibitors, SAHA and TSA, were used for comparison. Western blotting analysis was performed to investigate the acetylation of histone H3 and the expression of apoptotic markers in vitro and in vivo. Subcellular fractionation was performed to evaluate expression of Bax and cytochrome c in the cytosol and mitochondria, and also translocation of cytochrome c from the cytoplasm to the nucleus. A confocal microscopic evaluation was performed to confirm inhibition of cell proliferation, induction of apoptosis, and the nuclear translocation of cytochrome c in RCC cells.

**Results:**

In this study, we investigated the apoptosis-inducing activity of HNHA in cultured kidney cancer cells. Apoptosis in the HNHA-treated group was induced significantly, with marked caspase activation and Bcl-2 suppression in RCC cells in vitro and in vivo. HNHA treatment caused cytochrome c release from mitochondria, which was mediated by increased Bax expression and caspase activation. HNHA also induced nuclear translocation of cytochrome c, suggesting that HNHA can induce caspase-independent nuclear apoptosis in RCC cells. An in vivo study showed that HNHA had greater anti-tumor and pro-apoptotic effects on RCC xenografts than the established HDAC inhibitors.

**Conclusions:**

HNHA has more potent anti-tumor activity than established HDAC inhibitors. Its activities are mediated by caspase-dependent and cytochrome-c-mediated apoptosis in RCC cells. These results suggest that HNHA may offer a new therapeutic approach to RCC.

## Background

The initiation and progression of cancer, traditionally seen as a genetic disease, is now known to involve epigenetic abnormalities along with genetic alterations [1]. Epigenetic modifications, defined as heritable changes in gene expression that are *not* due to any alteration in the DNA sequence, play a key role in the regulation of all DNA-based processes, such as transcription, repair, and replication [2]. Consequently, abnormal expression patterns or genomic alterations in chromatin regulators have profound results and can lead to the development and maintenance of various cancer types [3].

One epigenetic modification common in several tumors is the modification of histones. Histones are the chief protein components of chromatin, acting not simply as spools around which DNA is coiled, but also as regulators of chromatin dynamics [4]. Because histone modifications are proposed to affect chromosome function, inappropriate histone modifications would be expected to result in dysregulation of cell growth, leading to neoplastic transformation or cell death [3–6]. The histone-modifying enzymes, histone acetyltransferases—which include histone deacetylases (HDACs) and histone methyltransferases (HMTs)—regulate these modification processes.

HDACs are important regulators of gene expression that remove acetyl groups from histones enzymatically. Numerous studies have demonstrated aberrant expression of HDACs in human tumors, and the expression levels of HDAC1, −5, and −7 serve as molecular biomarkers of tumor versus normal tissue. Moreover, in several cancer types, overexpression of individual HDACs correlates with significant decreases in both disease-free and overall survival [7–11]. Recent studies revealed that HDAC plays an important role in carcinogenesis and the overexpression of HDACs has been linked to key events in the repression of the tumor suppressor gene CDKN1A, encoding p21, and genes encoding DNA damage repair enzymes, such as BRCA1 and ATR [12].

Renal cell carcinoma (RCC) is a malignancy of the kidney that originates in the proximal renal tubule and accounts for ~3% of all cancers [13]. Although the incidence of small renal masses is high, approximately one in three patients presents with metastatic disease [14]. RCC is highly resistant to chemotherapy and radiotherapy; non-specific immunotherapy using interleukins and interferons are used as a standard treatment; however, the response rate is low.

Recent clarification of the molecular mechanisms of RCC has permitted tremendous progress in the development and approval of multiple targeted agents for the treatment of advanced RCC. Therapies targeted at the “vascular endothelial growth factor” (VEGF) and “mammalian target of rapamycin” (mTOR) pathways now represent the standard of care in metastatic RCC [13,14]. However, durable therapeutic responses to these therapies are uncommon, and the prognosis of RCC remains dismal.

Recent molecular investigations revealed that RCC has a higher percentage of global methylation and reduced histone acetylation, compared with non-tumor counterpart cells [15]. Several studies have demonstrated that histone deacetylases are highly expressed and decreased acetylation of histone H3 is a common alteration in RCC [16,17].

The use of massively parallel sequencing technologies enabled the discovery of additional common epigenetic modifications. Preclinical models have revealed that histone gene modifiers and epigenetic alterations may play important roles in RCC tumorigenesis and progression. *In vitro* analyses of renal tumor cell lines treated with vorinostat or ritonavir, alone and in combination, showed consistent dephosphorylation of Rb along with reduced HDAC1 expression and an increase in the sub-G1 fraction with combination treatment [18].

The biological outcome of HDAC inhibition is dependent on the HDAC specificity of the compound and the intrinsic operation of the cell-signaling pathways. Recently, N-hydroxy-7-(2-naphthylthio) hepatonomide (HNHA) has been introduced. It is a novel HDAC inhibitor that potently suppresses histone hypoacetylation and downregulation of HDAC target genes [19]. In this first study introducing HNHA, HNHA exhibited potent HDAC-inhibitory activity with IC50 values of 0.1 lM. In contrast, other compounds containing substituted nitrogen or oxygen for sulfur atom and having different lengths of alkyl chain (C5 and C7) showed weaker activity than that of HNHA. Moreover, compounds containing different zinc-chelating motifs such as an aminophenyl group or a hydroxy-acetylamino group had a weaker HDAC-inhibitory activity [19]. HNHA shows strong anti-cancer activity with pharmacological properties superior to those of the known HDAC inhibitor SAHA in human fibrosarcoma and breast cancer cells [19,20]. Here, we investigated this novel HDAC inhibitor and its mitochondrion-targeted actions in RCC and discuss the role of HNHA in induction of apoptotic cell death pathways in RCC.

## Methods

### Cell culture

The human RCC cell lines Caki-1 and A-498 were obtained from the American Type Culture Collection and maintained in McCoy’s 5A medium (Hyclone, Thermo Fisher Scientific), supplemented with 10% fetal bovine serum (FBS) and in minimal essential medium (MEM; Invitrogen), supplemented with 10% FBS and 2 mM L-glutamine. Cells were cultured at 37°C in a humidified atmosphere with 5% CO_2_. For all experiments, cell lines were maintained for no more than 2 months.

### Cell viability assay

Cell proliferation was measured using the MTT (3-(4,5-dimethylthiazol-2-yl)-2,5-diphenyltetrazolium bromide) assay. Cells were seeded in 96-well plates at 5 × 10^3^ per well and incubated overnight to 70% confluency. Drug was added at 0-120 μM final concentrations. After drug treatment, cells were incubated for 0, 12, 24, 36, 48, 60, and 72 h and cell viability was then measured using the MTT reagent at 490 nm according to the manufacturer’s protocol. Briefly, MTT reagent (10 μL) was added to each well (final concentration, 0.5 mg/mL) and the plate was incubated at 37°C. After 4 h, 100 μL of solubilization solution (10% SDS) were added to each well and the plate was incubated for 2 h. Data are presented as mean percentages of vehicle-treated cell proliferation ± SEM of triplicate experiments.

### TUNEL assay

To assay apoptosis, cells were fixed with 4% paraformaldehyde after 48 h of treatment and stained using a terminal deoxynucleotidyl transferase dUTP nick-end labeling (TUNEL) kit (Promega, Madison, WI). Apoptotic (fluorescent green) and total cells were counted under a fluorescence microscope and data were recorded. Images were obtained using a confocal microscope (LSM Meta 700, Carl Zeiss, Oberkochen, Germany) and analyzed using the Zeiss LSM Image Browser software (ver. 4.2.0121).

### Cellular fractionation

To assess cytochrome *c* release, cells were incubated with 0.05% digitonin in an isotonic sucrose buffer (250 mM sucrose, 10 mM HEPES-NaOH, 10 mM KCl, 1.5 mM MgCl_2_, 1 mM EDTA, 1 mM EGTA, and 0.5 mM phenylmethylsulfonyl fluoride; pH 7.2) at room temperature. At this concentration, digitonin permeabilizes the plasma membrane but not the mitochondrial membrane. The digitonin extract was collected as the cytosolic fraction for immunoblot analysis. To examine Drp1 translocation to mitochondria during apoptosis, cells were collected in ice-cold isotonic sucrose buffer by gentle scraping and homogenized using a Wheaton homogenizer. The homogenate was centrifuged (1000 *g*) to remove debris and nuclei; the supernatant was subjected to a further centrifugation step (10,000 *g*) to yield the mitochondrial fraction. All homogenization and centrifugation procedures were conducted at 4°C.

### Western blotting analysis

Cells were washed twice with cold phosphate-buffered saline and lysed on ice with RIPA buffer (Thermo Fisher Scientific, Suwanee, GA, USA) following the manufacturer’s protocol. Protein concentrations were determined by BCA assay (Pierce Biotechnology, Rockford, IL). Equal amounts of protein (20 μg) were separated in 8-10% SDS-polyacrylamide gels; the resolved proteins were then electro-transferred onto PVDF membranes (Millipore, Bedford, MA). The membranes were subsequently blocked with 5% nonfat milk in TBST for 1 h at room temperature and incubated with appropriate concentrations of primary antibodies—acetyl-histone H3 and histone H3 (Abcam, Cambridge, UK), acetyl-α-tubulin and α-tubulin (Abcam), Bax (Novus Biologicals, Littleton, CO, USA), p53 (Abcam), p21 (Abcam), Apaf-1 (Santa Cruz Biotechnology, Santa Cruz, CA, USA), CDK-4 (Cell Signaling Technology, Beverly, MA, USA), CDK-6 (Cell Signaling Technology), cyclin D1(Cell Signaling Technology), caspase-3 (Santa Cruz), caspase-7 (Santa Cruz), Bcl-2 (Santa Cruz), p-IκB (Abcam), cytochrome *c* (Abcam), and β-actin (Santa Cruz)—overnight at 4°C. The membranes were then washed three times with TBST and probed with the corresponding secondary antibodies conjugated to HRP (Santa Cruz) at room temperature for 1 h. After washing, blots were developed with ECL reagents (Pierce, Rockford, US) and exposed to Kodak X-OMAT AR Film (Eastman Kodak, Rochester, US) for 3 min.

### Human kidney cancer cell xenograft

Animals were maintained under specific pathogen-free (SPF) conditions. All experiments were approved by the Animal Experiment Committee of Yonsei University. Human kidney cancer cells (1.0 × 10^7^/mouse) were cultured *in vitro* and injected subcutaneously into the upper left flank region of the mice. The mice were maintained at 22°C with a 12/12-h light/dark cycle and access to food and water *ad libitum*. After 10 days, tumor-bearing mice were grouped randomly (*n* = 10/group) and an intraperitoneal injection of the three drugs (SAHA, TSA, and HNHA) was administered once every 2 days for a total of six injections (30 mg/kg per mouse) when the complete tumor size reached [4/3 × π × (0.7 × 0.4 cm)^3^ × ½]. Tumor size was measured every other day using calipers. Tumor volume was estimated using the following formula 4/3 × π × (a cm × b cm)^3^ × ½, where a and b are the two perpendicular diameters. SAHA, TSA, and HNHA doses were chosen based on published studies HDAC inhibitor [20–22].

### *In vivo* toxicity study

*In vivo* toxicity assays were performed using BALB/c nude mice. Six-week-old mice were caged for 1 week for acclimatization. Each group of 25 mice was injected intraperitoneally with SAHA, TSA, or HNHA at a dose of 30 mg/kg per mouse. The animals were monitored regularly for external signs of toxicity or lethality.

### Immunohistochemistry

All tissues were fixed in 10% neutral-buffered formalin and embedded in paraffin wax using standard protocols. Tissue sections (5 μm) were dewaxed and antigen retrieval was performed in citrate buffer (pH 6), using an electric pressure cooker set at 120°C for 5 min. Sections were incubated for 5 min in 3% hydrogen peroxide to quench endogenous tissue peroxidase. The primary monoclonal antibody was directed against cytochrome *c* (Abcam, Cambridge, UK), and was diluted 1:100with phosphate-buffered saline. All tissue sections were counterstained with hematoxylin, dehydrated, and mounted.

### Statistical analysis

Statistical analyses were performed using the GraphPad Prism software (GraphPad Software Inc., La Jolla, CA, USA). Immunohistochemistry results were subjected to one-way ANOVA followed by a Bonferroni *post hoc* test. Values are expressed as means ± SEM. P values < 0.05 were considered to indicate statistical significance.

## Results

### HNHA induced histone H3 acetylation in RCC cells

**Figure 1:**
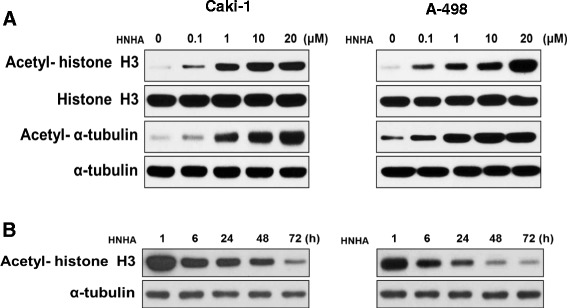
**HNHA induced acetylation of histone H3 in RCC cells.** Caki-1 and A-498 cells were treated for 24 h with 0.1, 1, 10, or 20 μM HNHA **(A)**. Caki-1 and A-498 were treated with HNHA (15 μM) for 1, 6, 24, 48, or 72 h **(B)**. Total proteins were isolated and histone H3 and α-tubulin acetylation was evaluated by Western blotting.

**Figure 2:**
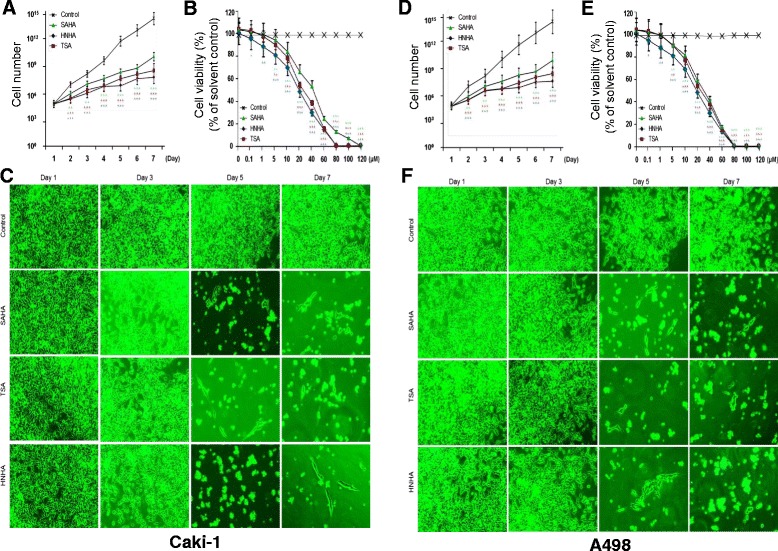
**HNHA suppressed RCC tumor cell proliferation.** Cell viability and cell adhesion assays showed that HNHA induced the greatest inhibition of tumor cell proliferation in Caki-1 **(A-C)** and A-498 cells **(D-F)** Points indicate mean % of the solvent-treated control. **(A, D)** and **(B, E)** experiments were repeated at least three times with similar results. *P < 0.05 vs. Control, **P < 0.01 vs. Control, ***P < 0.005 vs. Control.

To investigate the effects of HNHA on histone acetylation in RCC cells, we exposed Caki-1 and A-498 cells to HNHA at various doses and then evaluated histone H3 acetylation by Western blotting. Acetylation of histone H3 was induced by HNHA in a dose-dependent manner (Figure 1A). We also assessed the effects of HNHA on the acetylation of non-histone proteins using α-tubulin; α-tubulin acetylation was also increased by HNHA in a dose-dependent manner (Figure 1A). To determine the duration of maintenance of histone H3 acetylation by HNHA, protein levels were evaluated by Western blotting at 1, 6, 24, 48, and 72 h after HNHA treatment. Histone H3 acetylation peaked at 1 h after administration of HNHA and remained stable until 48 h (Figure 2B). These data show that HNHA can induce stable acetylation of histone H3, and also non-histone proteins, in RCC cells.

### HNHA suppressed RCC tumor cell proliferation

To investigate the antitumor effects of HNHA in comparison with previously established HDAC inhibitors in RCC, a cell proliferation assay using SAHA, TSA, and HNHA and a panel of RCC cell lines was performed (Table 1). Compared with SAHA and TSA, HNHA had a lower IC_50_ in all RCC cell lines (Caki-1, A-498, 786-O, UMRC-3) tested. To further characterize the effects of HNHA on RCC tumor cell viability, we evaluated Caki-1 and A-498 cells. All HDAC inhibitors (SAHA, TSA and HNHA) reduced the viability of RCC cells compared to controls. However, HNHA showed the strongest suppression of cell proliferation among the HDAC inhibitors (Figure 2A, Caki-1 and D, A-498); moreover, the suppression of proliferation was dose-dependent (Figure 2B, Caki-1 and E, A-498). Cell numbers and adhesion abilities were lower in the HNHA-treated groups than the control group, and the cells exhibited a more elongated shape, consistent with the morphological changes caused by SAHA and TSA treatment (Figure 2C, Caki-1 and F, A-498).

**Table 1:** **IC**_**50**_ **(half maximal inhibitory concentration) determination using a cell proliferation assay**

**Cell line**	**Tissue derived**	**Animal**	**Cell proliferation IC50 (μM)**		
			**HNHA**	**TSA**	**SAHA**
Caki-1	Kidney: clear cell type renal cell arcinoma	Human	20.61 (±0.9)	49.29 (±1.1)	32.14 (±1.1)
A498	Kidney; renal cell carcinoma	Human	20.95 (±1.0)	40.19 (±1.4)	33.01 (±1.8)
786-O	Kidney; clear cell type renal cell carcinoma	Human	23.29 (±1.1)	45. 30 (±1.0)	30.97 (±1.7)
UMRC-3	Kidney; clear cell type renal cell carcinoma	Human	32.28 (±1.0)	53.42 (±1.0)	40.13 (±0.7)

Each data point represents the mean of three independent IC_50_ experiments performed in triplicate by MTT assay. SD, standard deviation.

### HNHA induced G0/G1 cell cycle arrest in RCC cells

**Figure 3:**
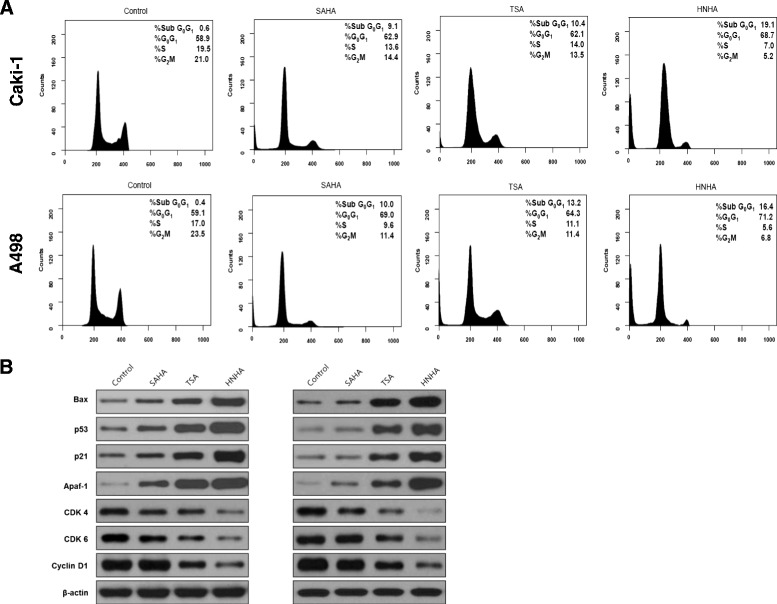
**HNHA induced G0/G1 phase arrest and apoptosis in RCC cells.** FACS flow cytometry with propidium iodide staining showed that HNHA induced G0/G1 phase arrest and increased the sub-G0 population in Caki-1 and A-498 cells **(A)**. Western blotting showed that HNHA potently induced the expression of cell cycle arrest proteins (p53 and p21), reduced positive regulators of cell cycle progression (CDK4/6 and cyclin D1) and resulted in increased levels of anti-apoptotic (Bax and Apaf-1) proteins in RCC cells **(B)**.

To investigate the effects of HNHA on cell cycle progression, propidium iodide staining and flow cytometry were performed after treatment with several HDAC inhibitors, including HNHA. The HDAC inhibitors induced G0/G1 phase arrest and increased the sub G0 population (p < 0.05), suggesting induction of cell cycle arrest and apoptosis in RCC (Figure 3A). The data also suggested that HNHA was the most potent apoptosis inducer of those tested. To assess pro-apoptotic signaling pathways following treatment with these drugs, we evaluated the expression of cell-cycle arrest (p53 and p21) and anti-apoptotic (Bax and Apaf-1) proteins by Western blotting (Figure 3B). HNHA resulted in increased levels of the cell-cycle arrest (p53 and p21) and anti-apoptotic (Bax and Apaf-1) proteins in RCC cells. Furthermore, HNHA reduced CDK 4, CDK 6 and cyclin D1, a positive regulator of the cell cycle, which is comparable to the highest inhibition of viability in RCC cells.

### HNHA-induced apoptosis of RCC cells was caspase-dependent

**Figure 4:**
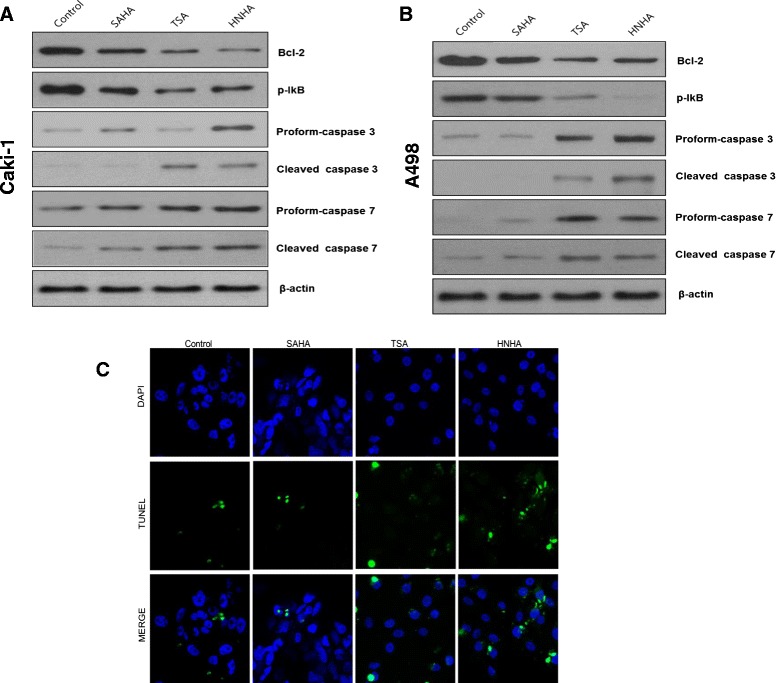
**HNHA induced caspase-dependent apoptotic death of RCC cells.** Immunoblot analysis for Bcl-2, phospho-IκB, and caspases-3 and -7 in Caki-1 and A-498 cells **(A, B)**. TUNEL assay of apoptotic cells in Caki-1 and A-498 cells; TUNEL-positive (apoptotic) cells are indicated (×400) **(C)**.

SAHA and TSA induce apoptosis by activating the caspase pathways in tumor cells. To investigate whether the antitumor effects of HNHA were dependent on pro-apoptotic signaling pathways, we evaluated Bcl-2 expression and the activation of caspases-3 and -7 in Caki-1 and A-498 cells treated with SAHA, TSA, and HNHA by Western blotting and TUNEL assays. Western blots showed that HNHA enriched the ‘pro’ form of caspase-3 and induced the cleavage of pro-caspase-3 and pro-caspase-7 more potently than did TSA or SAHA (Figure 4A, B). The TUNEL assay confirmed that HNHA induced apoptosis in RCC cells more potently than did TSA or SAHA (Figure 4C). Moreover, HNHA and SAHA abolished Bcl-2 and p-IκB expression almost completely in RCC cells. These data suggest that HNHA is the most potent apoptosis inducer of the compounds tested and exerts this effect via caspase activation and inhibition of the Bcl-2 pathway in RCC cells.

### HNHA induced cytochrome *c* release from mitochondria and translocation from the cytoplasm to the nucleus in RCC cells

**Figure 5:**
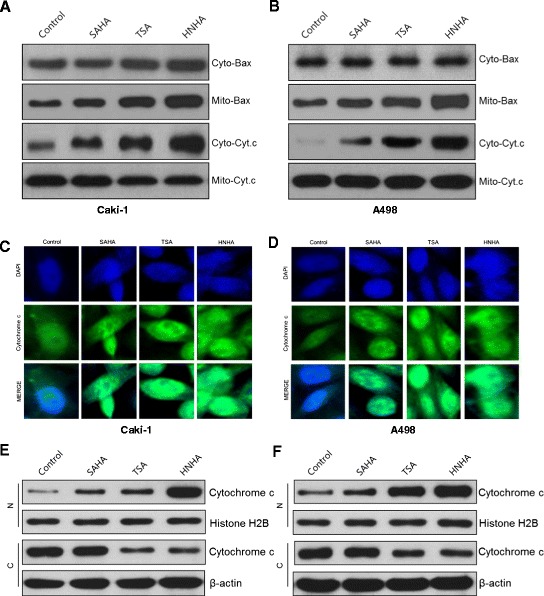
**HNHA induced cytochrome c-dependent, caspase-dependent apoptotic death in RCCs.** Subcellular fractionation showed that the Bax level was increased, and cytochrome c release into the cytosol was enhanced by HNHA, in Caki-1 **(A)** and A 498 cells **(B)**. Immunofluorescent cytochemical staining showed that cytochrome c was translocated and accumulated in the nucleus, suggesting that HNHA induced apoptosis through a cytochrome-c-dependent pathway. **(C, D)** Western blot analysis after subcellular fractionation confirmed that cytochrome c was translocated into the nucleus after HNHA treatment **(E, F)**.

DNA damage induces apoptosis by release of cytochrome *c* from mitochondria. To assess the mechanisms of HNHA-induced apoptosis, we next evaluated the expression of cytochrome *c* and caspases using immunofluorescence and Western blotting. Subcellular fractionation demonstrated that the HDAC inhibitors activated the mitochondrial pathway of apoptosis, characterized by the translocation of Bax (a proapoptotic Bcl-2 protein) from the cytosol to the mitochondria and the release of cytochrome *c* from the mitochondria into the cytosol in Caki-1 and A-498 cells (Figure 5A, B). Immunofluorescent cytochemical staining showed that cytochrome *c* was translocated and accumulated in the nucleus, suggesting that HNHA induces apoptosis through a cytochrome-*c*-dependent pathway (Figure 5C, D). Western blot analysis after subcellular fractionation that confirmed cytochrome *c* was translocated into the nucleus after HNHA treatment (Figure 5E, F). In summary, these results suggest that apoptosis is induced by HNHA through caspase- and cytochrome-*c*-dependent pathways in RCC cells.

### HNHA reduced the tumor burden and improved survival in RCC xenografts

**Figure 6:**
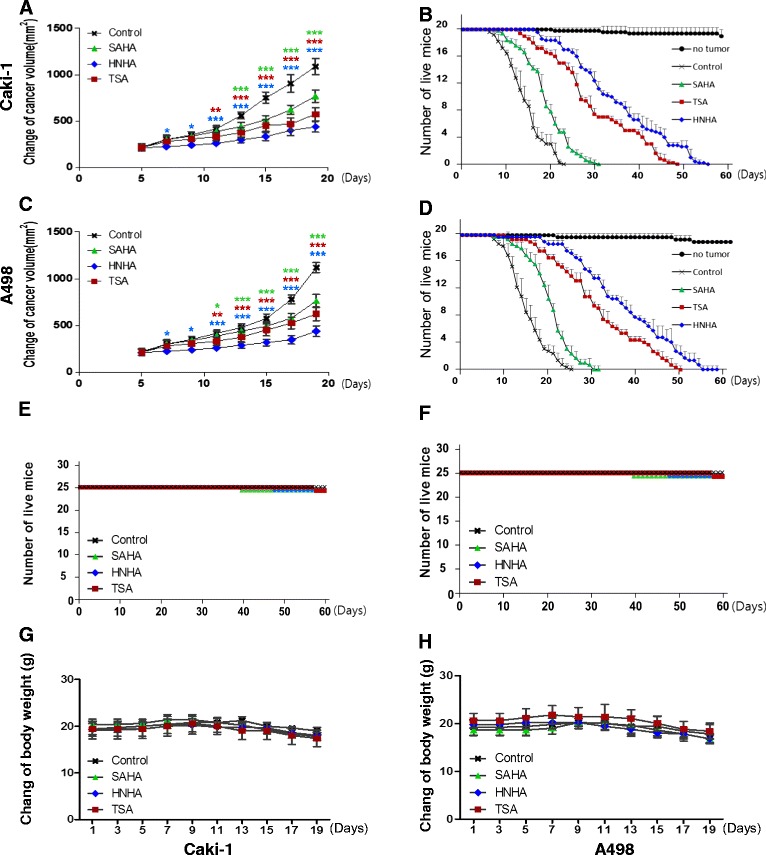
**HNHA exhibited the most potent anti-tumor effects in RCC xenografts.** HNHA induced more potent inhibition of tumor progression than the established HDAC inhibitors and resulted in the greatest prolongation of survival in Caki-1 **(A, B)** and A-498 **(C, D)** xenografts. ‘No tumor’ indicates HNHA-, TSA- and SAHA-treated mice without a xenograft in panels **(E)** and **(F)**. Each group = 10 mice, survival curves for 63 days. Mean body weights on days 1, 3, 5, 7, 9, 11, 13, 15, 17 and 19 of mice treated with HNHA, TSA and SAHA on Caki-1 **(G)** and A-498 **(H)** xenografts (Each group = 10 mice). *P < 0.05 vs. Control, **P < 0.01 vs. Control, ***P < 0.005 vs. Control.

To investigate the antitumor effect of HNHA *in vivo*, we developed subcutaneous RCC xenograft mouse models using Caki-1 and A-498 cells. SAHA and TSA showed significant suppression of tumor growth in RCC xenografts; however, HNHA exhibited greater suppression of the growth of RCC xenografts (Figure 6A, B). Also, survival was prolonged significantly by all HDAC inhibitors, but to a greater extent by HNHA than SAHA or TSA (Figure 6C, D). No evidence of systemic toxicity or treatment-related death was found in any group (Figure 6E, F). There was no significant effect on the body weight of mice treated with SAHA, TSA and HNHA compared to the control group (Figure 6G, H).

### HNHA suppressed *in vivo* tumor growth via downregulation of Bcl-2 and p-IκB and activation of caspase in RCC cells

**Figure 7:**
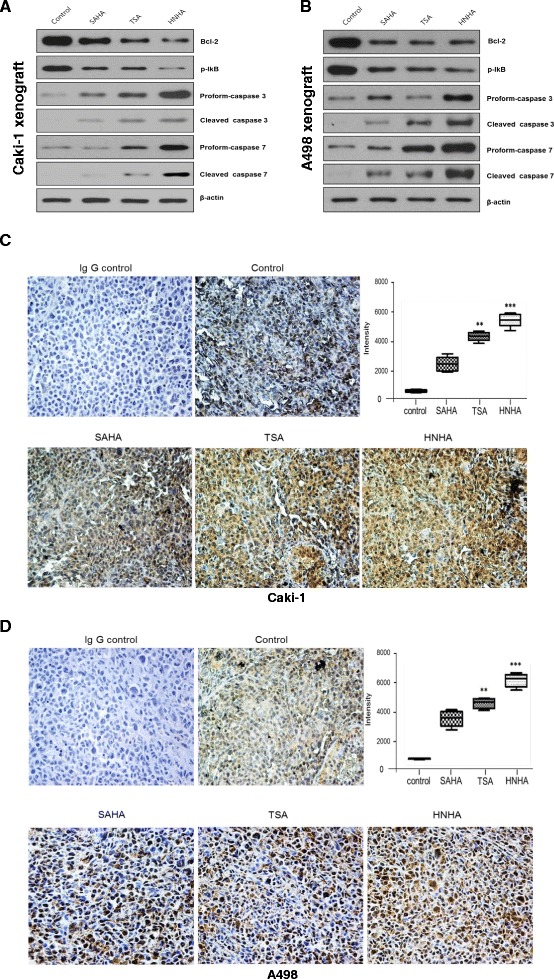
**HNHA reduced tumor burden and induced caspase-dependent apoptotic cell death in an** ***in vivo*** **model.** Immunoblot analysis for Bcl-2, phospho-IκB, and caspases-3 and -7 in Caki-1 and A-498 cells **(A, B)**. Cytochrome *c* protein levels were determined by immunohistochemistry to decide when to sacrifice the animal **(C, D)**. Negative control slides (IgG control) were treated with isotype-matched IgG. Error bars show SEs (*n* = 7). *P < 0.05, vs. control, **P < 0.01, vs. control, ***P < 0.005, vs. control. The MetaMorph 4.6 image-analysis software was used to quantify the immunostained target protein.

To determine whether the potent antitumor effect of HNHA in the *in vivo* animal model was related to the induction of apoptosis, we examined the expression of apoptosis-related proteins in xenograft tissues harvested after treatment. All HDAC inhibitors downregulated Bcl-2 expression and activation of IκB, induced the ‘pro’ forms of caspases-3 and -7, and activated caspases-3 and -7 by cleavage in Caki-1 and A-498 xenografts (Figure 7A, B). HNHA showed the most potent inhibition of Bcl-2 and p-IκB and activation of caspase in RCC cells. To examine whether the induction of apoptosis was related to cytochrome *c* release, we performed immunohistochemical staining for cytochrome *c* in the harvested xenografts (Figure 7C, D). Cytochrome *c* was stained focally in the cytoplasm in the control group but diffusely throughout the cytoplasm in the groups treated with HDAC inhibitors. The staining was most intense in the HNHA-treated xenografts. In summary, these data indicate that HNHA induced more potent RCC tumor suppression in an animal model by activation of caspase-dependent apoptotic signals and cytochrome *c* release from mitochondria.

## Discussion

One of the most remarkable advances in RCC treatment is the therapeutic application of anti-angiogenic therapy and mTOR inhibitors based on the important role of the HIF pathway in RCC. Most agents available today for the treatment of advanced RCC target the von Hippel-Lindau gene (VHL)/hypoxia-inducible factor (HIF) pathway [23]. Agents targeting the vascular endothelial growth factor (VEGF) and/or mammalian target of rapamycin (mTOR) pathways continue to be the mainstay of treatment for metastatic RCC; however, durable, complete responses remain the exception. There is a continuing need for the identification of novel pathways and agents for treating RCC [24].

Recent progress in understanding the molecular mechanisms in RCC centers on the roles of epigenetic alterations and transcriptional deregulation. A high-throughput gene study involving solution capture and sequencing of the coding exons of 20,000 protein-coding genes identified new cancer loci, including genes encoding enzymes that demethylate/methylate key lysine residues in histone H3, which is implicated in transcriptional control by regulating chromatin structure [23,25]. Another study found truncating mutations in the PBRM1 gene, which encodes the Baf180 protein, a chromatin-targeting subunit of the SWI/SNF chromatin remodeling complex that has been implicated in multiple chromatin/transcriptionally mediated processes through interaction with histone H3, in 41% of RCC cases [26].

Histone modification is now a well-known epigenetic modification. Among several types of histone modification, histone deacetylation is deregulated in many cancers. A recent study revealed that HDAC1, HDAC2, and HDAC3 are highly expressed in RCC [16]. Several studies point to overexpression of class I HDACs, in particular HDAC1, as a cancer marker associated with a poor prognosis [27]. HDAC inhibitors have been developed to reverse gene silencing by inhibiting HDAC activity and increasing histone acetylation. These inhibitors function by binding to the catalytic site of the enzyme. There are four distinct classes of HDAC inhibitor: short-chain fatty acids (valproic acid and butyrates), hydroxamic acids (trichostatin A, TSA, and suberoylanilide hydroxamic acid), cyclic tetrapeptides (trapoxin and depsipeptide), and benzamides [28,29].

Preclinical studies have shown the potential of HDAC inhibitors in the treatment of RCC. Valproic acid (VPA) altered cell-cycle-regulating proteins, particularly CDK2, cyclin B, cyclin D3, p21, and Rb, and significantly inhibited the growth of Caki-1 in subcutaneous xenografts, accompanied by strong accumulation of p21 and Bax in tissue specimens of VPA-treated animals [30]. Another HDAC inhibitor, SAHA, reduced the proliferation of RCC cells and augmented the activity of the mTOR inhibitor temsirolimus to induce apoptosis in xenografted RCC cell lines and depsipeptide-induced apoptosis in Caki-1, ACHN, and 786O cells, and induced G2 cell cycle arrest in 769P cells [17,31,32]. However, in contrast with the promising preclinical results, clinical trials of the HDAC inhibitors vorinostat and panobinostat have shown only modest activity in refractory metastatic RCC. In a phase II study of SAHA (vorinostat) in advanced RCC patients, SAHA showed disease stabilization in 29% patients, while a phase II trial of a different HDAC inhibitor, panobinostat, in patients with refractory metastatic RCC, failed to show an objective response [33,34]. It is difficult to evaluate the anti-tumor activity of these HDAC inhibitors for treatment-naïve advanced RCC because all participants in the study had previously been heavily treated. Nevertheless, that HDACs exist as targets and HDAC inhibitors show anti-tumor activities in RCC, but only modest activity in clinical trials, suggests that more potent HDAC inhibitors are required to achieve a clinical benefit in RCC.

In summary, we showed that HNHA had more potent anti-tumor activity than the established HDAC inhibitors SAHA and TSA with induction of apoptosis through expression of Bcl-2 and engagement of the mitochondrial pathway in RCC cells *in vitro* and *in vivo*. HNHA activated the caspase- and cytochrome-*c*-dependent apoptotic pathways by inducing release of cytochrome *c* from mitochondria and its translocation into the nucleus in RCC cells. In our study, Bax was found to be upregulated markedly by all HDAC inhibitors tested—albeit to a greater degree by HNHA than SAHA or TSA—in RCC cells. HNHA treatment resulted in the greatest level of cytochrome *c* release from mitochondria among the HDAC inhibitors tested.

Taken together, although the precise mechanisms of the anti-cancer effects of HDAC inhibition should be investigated further, our results suggest that HNHA may be a potent therapeutic option. The role of HDAC inhibition as an anti-cancer strategy in RCC should be evaluated using agents more potent than those tested previously.

## Conclusions

HNHA has more potent anti-tumor activity than established HDAC inhibitors. Its effects are mediated by caspase-dependent and cytochrome-c-mediated apoptosis in RCC cells. These results suggest that HNHA may offer a new therapeutic approach to RCC.
